# Prediction of brain age using quantitative parameters of synthetic magnetic resonance imaging

**DOI:** 10.3389/fnagi.2022.963668

**Published:** 2022-11-15

**Authors:** Shasha Bao, Chengde Liao, Nan Xu, Ailin Deng, Yueyuan Luo, Zhiqiang Ouyang, Xiaobin Guo, Yifan Liu, Tengfei Ke, Jun Yang

**Affiliations:** Department of Radiology, Yunnan Cancer Hospital/Center, Third Affiliated Hospital of Kunming Medical University, Kunming, China

**Keywords:** brain age prediction, synthetic MRI, relaxation value, brain aging, brain regions

## Abstract

**Objective:**

Brain tissue changes dynamically during aging. The purpose of this study was to use synthetic magnetic resonance imaging (syMRI) to evaluate the changes in relaxation values in different brain regions during brain aging and to construct a brain age prediction model.

**Materials and methods:**

Quantitative MRI was performed on 1,000 healthy people (≥ 18 years old) from September 2020 to October 2021. T1, T2 and proton density (PD) values were simultaneously measured in 17 regions of interest (the cerebellar hemispheric cortex, pons, amygdala, hippocampal head, hippocampal tail, temporal lobe, occipital lobe, frontal lobe, caudate nucleus, lentiform nucleus, dorsal thalamus, centrum semiovale, parietal lobe, precentral gyrus, postcentral gyrus, substantia nigra, and red nucleus). The relationship between the relaxation values and age was investigated. In addition, we analyzed the relationship between brain tissue values and sex. Finally, the participants were divided into two age groups: < 60 years old and ≥ 60 years old. Logistic regression analysis was carried out on the two groups of data. According to the weight of related factors, a brain age prediction model was established and verified.

**Results:**

We obtained the specific reference value range of different brain regions of individuals in different age groups and found that there were differences in relaxation values in brain tissue between different sexes in the same age group. Moreover, the relaxation values of most brain regions in males were slightly higher than those in females. In the study of age and brain relaxation, it was found that brain relaxation values were correlated with age. The T1 values of the centrum semiovale increased with age, the PD values of the centrum semiovale increased with age, while the T2 values of the caudate nucleus and lentiform nucleus decreased with age. Seven brain age prediction models were constructed with high sensitivity and specificity, among which the combined T1, T2 and PD values showed the best prediction efficiency. In the training set, the area under the curve (AUC), specificity and sensitivity were 0.959 [95% confidence interval (CI): 0.945–0.974], 91.51% and 89.36%, respectively. In the test cohort, the above indicators were 0.916 (95% CI: 0.882–0.951), 89.24% and 80.33%, respectively.

**Conclusion:**

Our study provides specific reference ranges of T1, T2, and PD values in different brain regions from healthy adults of different ages. In addition, there are differences in brain relaxation values in some brain regions between different sexes, which help to provide new ideas for brain diseases that differ according to sex. The brain age model based on synthetic MRI is helpful to determine brain age.

## Introduction

With the aging of the global population, the burden of age-related functional decline and disease is increasing (Vos et al., 2012). As one’s age increases, the microscopic and macroscopic characteristics of white matter change, and the atypical manifestations of white matter in the process of aging are similar to neurodegenerative diseases and mental diseases (Valizadeh et al., 2018). Therefore, understanding the brain-related changes in the process of aging can help us to better distinguish them from signs of a pathological state (Lebel and Deoni, 2018). There are considerable individual differences in the size of brain changes with age; the degree of brain aging may be adversely affected by poor physical and mental health. Cardiovascular risk factors, such as hypertension high blood pressure, diabetes and obesity, have been linked to increased brain aging (Ronan et al., 2016). In addition, some studies have hypothesized that long-term exposure to a stressful environment will lead to biological aging and premature aging (Geronimus, 2013). Therefore, in the process of human brain degeneration with age, the speed and trajectory of human brain degradation may be quite different between different individuals and different brain regions. Magnetic resonance imaging (MRI) is a powerful tool for studying human brain maturation, because many ongoing microstructure changes during brain maturation affect proton relaxation, leading to characteristic changes in MRI findings (Paus et al., 2001).

MRI features could serve as a biomarker for determining the age of the brain and can reflect the integrity and health of the brain to a certain extent (Bashyam et al., 2020). At present, a variety of brain MR quantitative methods, such as magnetization transfer imaging and diffusion tensor imaging, have been used to evaluate the physiological characteristics of brain tissue and its changes with age (Weiskopf et al., 2013; Seiler et al., 2014; Faizy et al., 2018). However, the traditional quantitative relaxation sequence needs to be collected on the basis of the conventional scanning sequence, which increases the scanning time. Different scanning sequence images may have the problem of spatial mismatching. Synthetic MRI (SyMRI) is a new type of quantitative MR technology that can obtain high-definition quantitative values of R1 (1/T1), R2 (1/T2) and proton density (PD) of the whole brain in a single scan with high accuracy, and the quantitative parameters related to brain tissue can be obtained according to the automatic segmentation of brain tissue by R1, R2 and PD (McAllister et al., 2017). In contrast, quantitative MRI techniques, such as relaxation measurements provide absolute digital indicators that can be used to define brain aging trajectories and quantitatively monitor abnormal aging or disease conditions.

SyMRI can not only provide the images needed for conventional imaging diagnosis, but also provide quantitative values for the early detection of abnormal maturity and disease status (Hagiwara et al., 2017). By drawing the region of interest (ROI) on the quantitative map of syMRI, the quantitative values of brain tissue can be obtained. Therefore, syMRI can objectively compare brain states according to the relaxation value and the absolute value of water content (Warntjes et al., 2008; Hagiwara et al., 2017). To date, the gradual changes in the contrast of gray matter and white matter on conventional images driven by changes in T1 and T2 values have been widely used to evaluate brain development (Barkovich et al., 1988; Paus et al., 2001). Compared with traditional MRI, syMRI significantly improves the examination efficiency, enriches diagnostic information, and has broader applicability. SyMRI technology can synthesize a variety of contrast-weighted images by setting different repetition time (TR), echo time (TE) and inversion time (TI) values, including some rarely used clinical scanning sequences, to provide more valuable diagnostic information for a variety of clinical diseases, and show a good diagnostic effect (Andica et al., 2019). At present, some studies have used the above characteristics of syMRI to evaluate the brain volumes of children with cerebral palsy (Kułak et al., 2016), autism (Hazlett et al., 2017) and multiple sclerosis brain atrophy (Vågberg et al., 2013). To sum up, we speculate that it is feasible to use syMRI to explore the brain aging process.

Previous studies have shown that T1, T2 and PD values obtained by using syMRI varied with age in 89 healthy children (from newborns to adolescents) (Lee et al., 2018). They also provided age-specific regional reference values that can be used as an objective tool for assessing normal/abnormal brain development (Lee et al., 2018). However, there are few studies on the changes in brain tissue relaxation values from adulthood to old age. The purpose of this study was to prospectively analyze the relevant information of relaxation values obtained by synthetic MRI in different brain regions of healthy people, explore the changes in brain tissue relaxation values in relation to age, and provide digital indicators, that can be used to define the aging trajectory to quantitatively monitor the status of abnormal aging.

## Materials and methods

### Subjects and groups

We recruited healthy volunteers from communities and universities, and all subjects underwent syMRI at our research center (September 2020 to October 2021, Asian population). Inclusion criteria were as follows: (1) age ≥ 18 years old; (2) no previous history of hypertension, heart disease or diabetes; (3) no severe mental illness, learning disability or cognitive impairment in the past; (4) no history of alcohol abuse or taking psychotropic drugs; (5) no history of brain-related lesions (such as cerebral hemorrhage, cerebral infarction, brain tumor, etc.) or history of brain surgery; and (6) no claustrophobia. Exclusion criteria were as follows: (1) MRI detection of brain tumors, cerebral hemorrhage and other brain abnormalities and (2) images with artifacts. We included 1,000 subjects, of whom 57 were diagnosed with old cerebral infarction, significant demyelination and severe motor artifacts, and were excluded from the final analysis. This study was examined and approved by the Ethics Committee of Yunnan Cancer Hospital (Ethical Review approval No: KYCS2021262). All participants were informed of the contents and methods of the experiment and signed an informed consent form.

### Magnetic resonance imaging scanning protocol and data preprocessing

All MRI scans were performed on a 3T scanner (GE Signal Pioneer, GE Medical) using a 21-channel head and neck joint coil. The quantitative MRI parameters were as follows: visual field 240 mm, matrix 320 × 256, cross-sectional thickness/spacing 6/1 mm, echo chain length 16, acceleration factor 3, excitation times 1, layer number 21, bandwidth 173.6 kHz/pixel, and TR 4,031 ms. There was a 1 mm spacing between slices. The main purpose of adding layer spacing is to prevent interlayer interference and to control the number of scanning layers. Too many layers will prolong the scanning time of Magic sequence and increase the probability of motion artifacts. The acceleration is ASSET (Array Spatial Sensitivity Encoding Technique. ASSET is GE’s rebranding of SENSE.). We use 4 different saturation delay times and 2 different echo times. The syMRI sequence acquisition time was 3 min 30 s. Use the program provided by the supplier (GE’s MAGIC software was used) to generate quantified maps (T1, T2 and PD diagrams) from the original data at the same time. MAGIC is a customized version of SyMRI IMAGE marketed by Synthetic MRI’s partner GE Healthcare under a license agreement.

### Brain tissue value measurement

We measured and analyzed the quantitative tissue values (T1, T2, and PD) of the ROI by the same surveyor. Three quantitative relaxation values were simultaneously obtained in each ROI by using imaging processing software (GE’s MAGIC software was used). The ROIs on T1 were manually placed in the following 17 regions of interest: the cerebellar cortex, pons, amygdala, hippocampal head, hippocampal caudate, temporal lobe, occipital lobe, frontal lobe, caudate nucleus head, lentiform nucleus shell, dorsal thalamus, centrum semiovale, parietal lobe, precentral gyrus, and postcentral gyrus. The substantia nigra and red nucleus were located on T2 fluid-attenuated inversion recovery (FLAIR) images. The selection of the ROI for quantitative analysis is shown in the supplementary (Supplementary Figure 1). The tissue values we obtained from the left and right hemispheres of the brain were averaged to obtain the value of each ROI in the brain. ROI delineated slice selection was performed at the (1) internal auditory canal level: cerebellar hemisphere; (2) middle layer of the eyeball: pons; (3) suprasellar cistern level: amygdala, hippocampal head, hippocampal caudate, temporal lobe, and occipital lobe; (4) basal ganglia level: frontal lobe, caudate nucleus head, lentiform nucleus shell, and dorsal thalamus; (5) centrum semiovale level: centrum semiovale, and parietal lobe; (6) paracentric lobule level: precentral gyrus, postcentral gyrus; and (7) midbrain aqueduct plane (on T2 FLAIR): substantia nigra and red nucleus. ROIs were chosen carefully to prevent the risk of partial volume effects at the tissue interface and to prevent changes in relaxation values from being drawn in the neighbor’s sulcus in smaller areas. Hence, the areas we manually delineated were located in the median area of the corresponding brain region to ensure that the values come from brain tissue, not from the nearest neighbor’s cerebrospinal fluid. Two weeks after the first drawing of the ROIs, we randomly selected 10 subjects (a total of 60 subjects) in each of the six groups, and the same radiologist drew the ROIs again. Then, the tissue values extracted twice were tested by the intragroup correlation coefficient test (intraclass correlation coefficient, ICC). The characteristics with ICCs > 0.75 were considered to be stable and repeatable.

### Statistical analysis

SPSS 22.0 software was used for statistical analysis in this study. Visual histogram inspections were used to assess variable normality. When normally distributed, a consistency test (ICC) was used to evaluate the intragroup differences in measurements from the same radiologist. The brain regions with ICC values > 0.75 were preserved, and the following statistical analysis was performed: (1) the analysis of variance (ANOVA) test or nonparametric test was used for comparisons among different age groups; (2) the independent sample *t*-test was used for comparison between different sexes in the same age group; (3) the values of T1, T2, PD of brain tissue were analyzed by Spearman correlation analysis, and the difference was evaluated for statistical significance; (4) the data were divided into two groups (< 60 years old group and ≥ 60 years old group) and analyzed by logistic binary regression analysis, and the brain regions with *p* < 0.05 were retained. All patients were randomly divided into a training set and a test set at a ratio of 7:3 (the division between test and training data based on the entire pool of subjects). RStudio (version 4.1.0) software was used to build the prediction model and verification model.

## Results

Among the 943 subjects, there were 357 males and 586 females aged from 20 to 85 years old. According to their age, the participants were divided into 6 groups: 350 were in the 20-year-old group, 70 were in the 30-year-old group, 110 were in the 40-year-old group, 170 were in the 50-year-old group, 132 were in the 60-year-old group and 111 were in the ≥ 70-year group. Figure 2 shows the age range and sex distribution of subjects. The brain regions with good consistency (ICC > 0.75) were retained when the observers measured the tissue values of each brain region twice (Supplementary Table 1).

**FIGURE 1:**
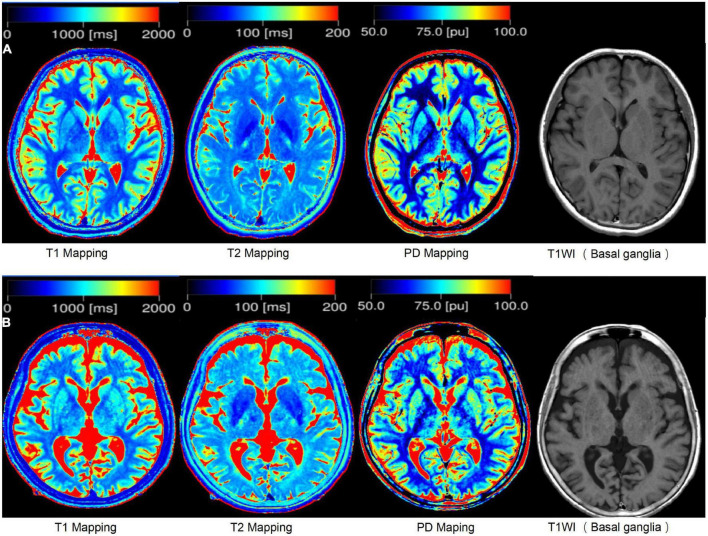
The example of syMRI. **(A)** Shows the brain of a 25-year-old person, and **(B)** shows the brain of a 83-year-old person.

**FIGURE 2:**
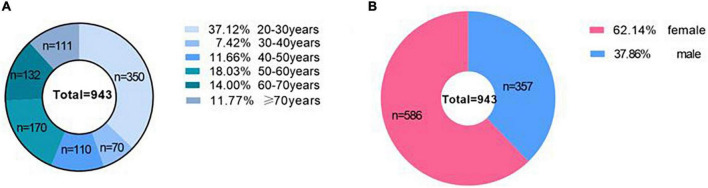
The age range and sex distribution of subjects. **(A)** Shows the age range of subjects, and **(B)** shows the sex distribution of subjects.

1. Comparison of brain tissue relaxation parameters between participants of the same age but of different sexes.

**FIGURE 3:**
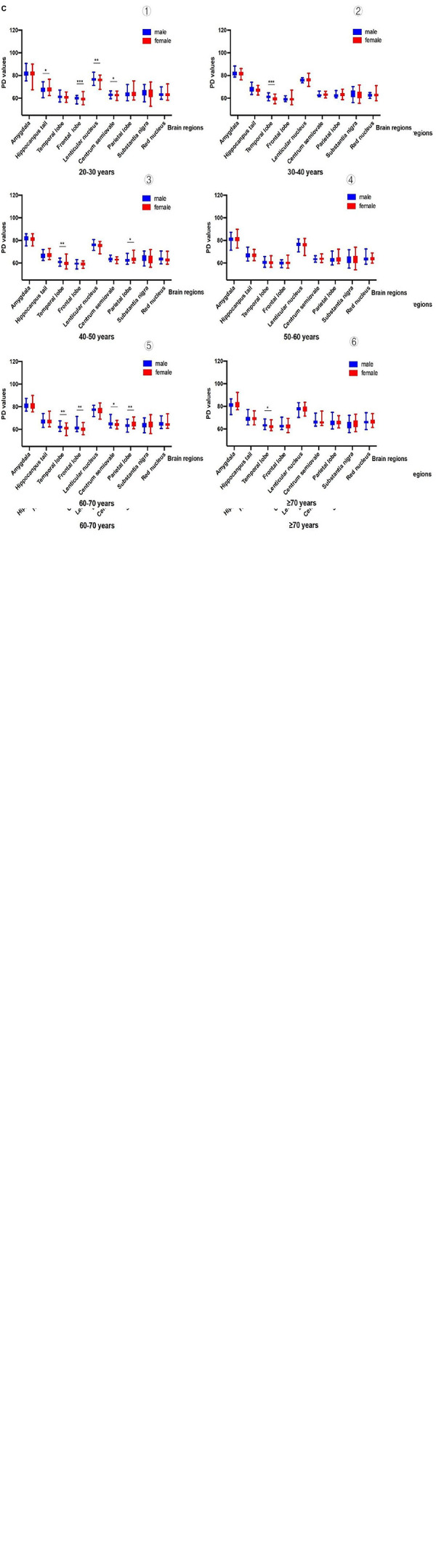
**(A)** Comparison of T1 values among people of different sexes [➀ shows sex differences in T1 values (20-30 years): in the frontal lobe, caudate nucleus, lentiform nucleus, centrum semiovale and substantia nigra; ➁ shows sex differences in T1 values (30-40 years): only in the frontal lobe; ➂ shows sex differences in T1 values (40-50 years):only in the substantia nigra; ➃ shows sex differences in T1 values (50-60 years): only in the frontal lobe; ➄ shows sex differences in T1 values (60-70 years): only in the frontal lobe; ➅ shows sex differences in T1 values (70 years): only in the frontal lobe]. (Male vs. female, *P<0.05; ^**^P<0.01; ^***^P<0.001). **(B)** Comparison of T2 values among people of different sexes [➀ shows sex differences in T2 values (20-30 years): in the caudate nucleus, lenticular nucleus, and parietal lobe; ➁ shows sex differences in T2 values (30-40 years):all brain regions had no significant difference; ➂ shows sex differences in T2 values (40-50 years): only in the hippocampal tail; ➃ shows sex differences in T2 values (50-60 years): only in the pons; ➄ shows sex differences in T2 values (60-70 years): all brain regions had no significant difference; ➅ shows sex differences in T2 values (70 years): all brain regions had no significant difference]. (Male vs. female, *P<0.05; ^**^P<0.01;^***^P<0.001). **(C)** Comparison of PD values among people of different sexes [➀ shows sex differences in PD values (20-30 years): in the hippocampal tail, frontal lobe, lentiform nucleus, and centrum semiovale; ➁ shows sex differences in PD values (30-40 years): only in the temporal lobe; ➂ shows sex differences in PD values (40-50 years): only in the temporal lobe and parietal lobe; ➃ shows sex differences in PD values (50-60 years): there was no significant difference in all brain regions; ➄ shows sex differences in PD values (60-70 years): in the temporal lobe, frontal lobe, centrum semiovale, and parietal lobe; ➅ shows sex differences in PD values (70 years): only in the temporal lobe]. (Male vs. female, *P<0.05;^**^P<0.01;^***^P<0.001).

After analyzing and comparing the relaxation values of brain tissue of participants within the same age group of different sexes, we found that there were significant differences in some brain regions between the sexes (Figure 3 and Supplementary Table 2).

The age groups and brain regions that showed sex differences in T1 values were (1) the 20–30 years old group: in the frontal lobe, caudate nucleus, lentiform nucleus, centrum semiovale and substantia nigra; (2) the 30–40 years old group: only in the frontal lobe; (3) the 40–50 years old group: only in the substantia nigra; (4) the 50–60 years old group: in the frontal lobe only; (5) the 60–70 years old group: in the frontal lobe only; and (6) the ≥ 70 years old group: in the frontal lobe only. The age groups and brain regions that showed sex differences in T2 values were (1) the 20–30 years old group: in the caudate nucleus, lenticular nucleus, and parietal lobe; (2) the 30–40 years old group: all brain regions had no significant difference; (3) the 40–50 years old group: only in the hippocampal tail; (4) the 50–60 years old group: only in the pons; (5) the 60–70 years old group: all brain regions had no significant difference; and (6) the ≥ 70 years old group: all brain regions had no significant difference. The age groups and brain regions with sex differences in PD values were (1) the 20–30 years old group: in the hippocampal tail, frontal lobe, lentiform nucleus, and centrum semiovale; (2) the 30–40 years old group: only in the temporal lobe; (3) the 40–50 years old group: only in the temporal lobe and parietal lobe; (4) the 50–60 years old group: there was no significant difference in all brain regions; (5) the 60–70 years old group: in the temporal lobe, frontal lobe, centrum semiovale, and parietal lobe; and (6) the ≥ 70 years old group: only in the temporal lobe.

2. Changes in the relaxation values of brain regions with age.

**FIGURE 4:**
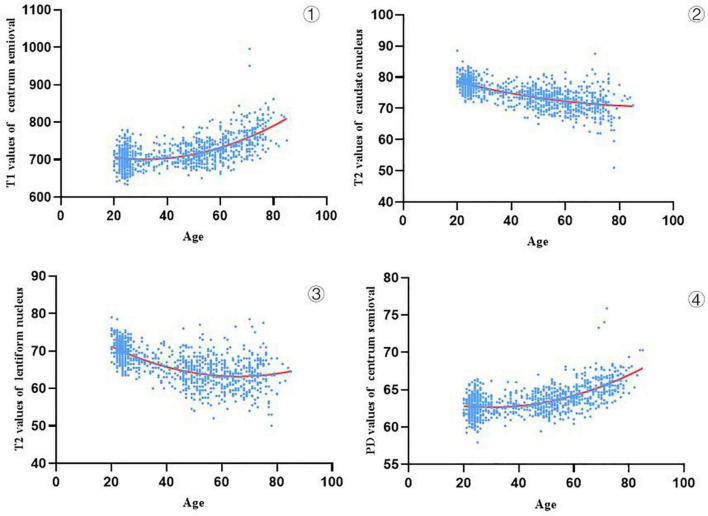
Trajectories of T1, T2 and PD values in age-related brain regions (943 subjects) **①** show that the T1 values of the centrum semiovale increased with age, **②,③** show that the T2 values of the caudate nucleus and lentiform nucleus decreased with age, and **④** show that the PD values of centrum semiovale increased with age.

The specific reference ranges of relaxation values for different brain regions (T1, T2, and PD) for different age groups are provided in Supplementary Table 3. After Spearman correlation analysis was performed between relaxation values and age, we selected the brain regions with significant correlation coefficients (| *r*| > 0.4, see Supplementary Table 4). Categorized by values, these regions were T1: the centrum semiovale; T2: the caudate nucleus and lenticular nucleus; and PD: centrum semiovale. Figure 4 shows the changes in T1, T2, and PD values with age in 943 subjects with higher correlation coefficients. We found that the relaxation values of brain regions with a high correlation with age had different trends with increasing age, in which the T1 values of the centrum semiovale increased with age, the PD values of centrum semiovale increased with age, while the T2 values of the caudate nucleus and lentiform nucleus decreased with age.

3. Construction and prediction of the prediction model.

We divided the included data into two groups, a < 60-year-old group (700 cases) and a ≥ 60-year-old group (243 cases), to build a brain age prediction model. We screened out the variables that entered the model by logistic binary regression to construct a brain age prediction model and finally included the brain regions corresponding to certain T1, T2, and PD values in the model as follows: (1) T1 values: the caudate nucleus, lenticular nucleus, centrum semiovale, substantia nigra and red nucleus; (2) T2 values: the frontal lobe, caudate nucleus, lenticular nucleus and dorsal thalamus; and (3) PD values: the centrum semiovale and red nucleus (Supplementary Table 5).

**FIGURE 5:**
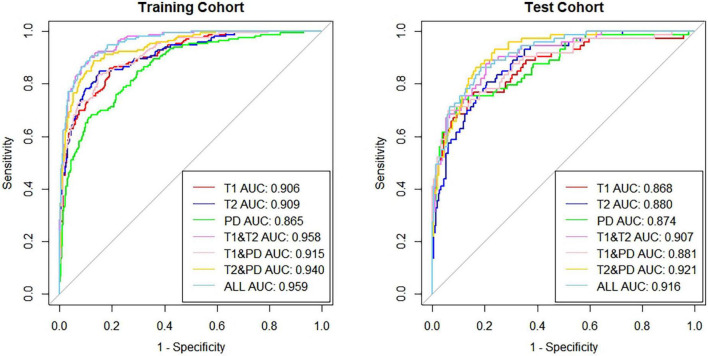
The AUC of the 7 predictive models in the training group and test group.

There are seven prediction models: (1) the T1 value prediction model; (2) the T2 value prediction model; (3) the PD value prediction model; (4) the T1 value combined with the T2 value prediction model; (5) the T1 value combined with the PD value prediction model; (6) the T2 value combined with the PD value prediction model; and (7) the T1 value, T2 value, and PD value joint prediction model. The receiver operating characteristic (ROC) curves of the training set and verification set of the seven models we constructed are shown in Figure 5. The characteristics of training cohort and test cohort are shown in Table 1. The sensitivity and specicity of each model are shown in Table 2.

**TABLE 1:** Demographic characteristic of subjects in training and test cohort.

	Training cohort (659 cases)	Test cohort (284 cases)	*P-value*
<60 years	489 (74.2%)	211 (74.3%)	—
≥60 years	170 (34.8%)	73 (25.7%)	—
Caudate nucleus T1	1017.29 ± 1.92	1022.31 ± 2.71	0.14
Lenticular nucleus T1	932.16 ± 1.99	930.57 ± 3.22	0.67
Centrum semiovale T1	721.38 ± 1.44	716.38 ± 2.38	0.06
Substantia nigra T1	650.79 ± 1.48	647.86 ± 2.17	0.27
Red nucleus T1	699.14 ± 1.30	699.88 ± 2.14	0.76
Frontal lobe T2	73.95 ± 0.13	73.45 ± 0.21	0.04
Caudate nucleus T2	74.48 ± 0.16	75.16 ± 0.23	0.02
Lenticular nucleus T2	66.13 ± 0.18	66.69 ± 0.27	0.09
Dorsal thalamus T2	71.65 ± 0.13	71.55 ± 0.22	0.68
Centrum semiovale PD	63.69 ± 0.07	63.41 ± 0.12	0.03
Red nucleus PD	63.93 ± 0.10	63.80 ± 0.14	0.45

**TABLE 2:** Specificity and sensitivity of 7 prediction models.

	Training cohort			Test cohort		
		
	*ROC*	*Specificity*	Sensitivity	ROC	*Specificity*	Sensitivity
T1	0.906 (0.881–0.931)	86.79%	85.96%	0.868 (0.816–0.921)	86.32%	82.00%
T2	0.909 (0.884–0.935)	85.87%	85.98%	0.880 (0.837–0.923)	84.39%	76.60%
PD	0.865 (0.833–0.8961)	82.75%	83.53%	0.874 (0.824–0.924)	85.19%	90.24%
T1/T2	0.958 (0.944–0.973)	89.94%	88.64%	0.907 (0.868–0.945)	87.34%	80.00%
T1/PD	0.915 (0.891–0.939)	87.07%	82.69%	0.881 (0.833–0.930)	87.07%	82.69%
T2/PD	0.940 (0.921–0.960)	89.27%	88.28%	0.921 (0.888–0.953)	87.28%	78.57%
T1/T2/PD	0.959 (0.945–0.974)	91.51%	89.36%	0.916 (0.882–0.951)	89.24%	80.33%

## Discussion

Based on syMRI of 943 healthy people aged 20–85 years old, this study analyzed the sex differences in the relaxation values of different brain regions and their changes with age.

In this study, syMRI was used for the first time to assess the developmental trajectory of brain tissue value attributes from adulthood to old age in a large sample. We analyzed the relationship between the relaxation values of different brain regions and age. The T1 values of the centrum semiovale increased with age, the PD values of the centrum semiovale increased with age, while the T2 values of the caudate nucleus and lentiform nucleus decreased with age. In addition, we established seven prediction models using brain tissue relaxation values to predict brain age, among which the model combined with T1, T2, and PD values had the best performance for brain age prediction. In this study, when exploring the relationship between brain tissue relaxation values and age, we also found that there may be differences in brain tissue relaxation values between different sexes. We found that the relaxation values of brain regions of participants in the same age group were different between different sexes, and the T1 values of brain regions with differences in males were slightly higher than those in females. However, the T2 and PD values of different brain regions in females were slightly higher than those in males.

In our study, we found that the differences in T1, T2, and PD values in different brain regions were different among different age groups, suggesting that the changes in relaxation values in important brain regions in different age groups may have stages. In the research progress on the structural changes in the cerebral hemisphere of aging rhesus monkeys, it has been proven that the decrease in oligodendrocyte and synaptic density are related to age; in addition, with the increase in age, glial cell proliferation, increase in free water content and decrease in myelin sheath leads to the increase in T1 and T2 values. These physiological changes affect the changes in white matter volume and relaxation value and have a certain correlation with age (Peters and Kemper, 2012). We found that there was a linear relationship between the relaxation value of brain tissue and age with increasing age. It was observed that some brain regions showed positive correlations, while some brain regions showed negative correlations. However, a study of newborns to adolescents showed that tissue values in all brain regions except cortical PD decreased with age (Lee et al., 2018). This finding is different from our results, which showed that the T1 and PD values of the centrum semiovale decrease with age, while the T2 values of the caudate nucleus and lenticular nucleus increase with age. This outcome may be due to complex biochemical and biophysical changes. In the study of healthy children (Lee et al., 2018), the brain develops to maturity from infancy to adolescence, while our subjects studied the aging process of the brain from adulthood to old age. The general decline in tissue value during brain maturation from infancy to puberty is due to complex biochemical and biophysical changes, such as reduced tissue water content, changes in water zoning, myelin formation, and changes in cell and axon density (Yoshiura et al., 1998, 2000; Matsumae et al., 2001; MacKay et al., 2006; Bartzokis et al., 2010; Glasser and Van Essen, 2011; Deoni et al., 2012). The physiological process of brain maturation is different from that of brain aging, so our results are different from those of teenagers.

Our study found that the T1 value of the centrum semiovale increased with age, which may be related to myelin loss, amyloid burden and water content. The loss of myelin and the increase in water content in brain tissue led to an increase in T1 values (Peters and Kemper, 2012). Myelin formation increases dramatically in the first few years of life (MacKay et al., 1994). At the same time, in the absence of pathology, the water content of brain tissue decreases in childhood (Kreis et al., 1993). In contrast, in the elderly stage, MRI provides evidence consistent with myelin pallor, diffuse vacuolation, gliosis, perivascular space dilation, and decreased glial cell density (Davis et al., 1994). These results are consistent with our findings obtained by syMRI in the investigation of biological markers of brain age. The T1 value of brain tissue from infants to adolescents (brain maturity) decreases with age (Lee et al., 2018). However, during brain aging, the T1 value of the centrum semiovale increased with age. The T2 value is related to amyloid deposition, myelin density, iron load and tissue water content (Schenker et al., 1993; Gracien et al., 2016). In addition, the accumulation of iron leads to the shortening of T2, especially for gray matter nuclei (Schenker et al., 1993). PD provides information about water content in tissue, so when brain tissue is replaced by cerebrospinal fluid, it is sensitive to edema and atrophy (Engström et al., 2014). With increasing age, the water content of brain tissue increases and brain atrophy occurs, so the PD values of the centrum semiovale is positively correlated with age.

Jackson et al. (1993) reported that the changes in brain tissues in different regions were the same, and there was a relative dividing line at approximately 60 years of age. Before 60 years of age, T1, T2, and PD values were stable or slightly decreased but increased after 60 years of age; this outcome may be due to changes in water, myelin sheath and iron content. In this study, we proposed to construct a model to predict the brain age of individuals < 60 years old and ≥ 60 years old. The seven prediction models we constructed have high prediction efficiency, and compared with the prediction of a single relaxation value, we found that the prediction model constructed by combining T1, T2, and PD values was the most effective. This model may be the most effective because the combination of multiple values may make the prediction information richer, thus improving the prediction accuracy and efficiency of the model. This model may be a suitable biological marker for predicting brain aging, which can aid in the early clinical diagnosis and early prevention of neurodegenerative diseases such as Alzheimer’s disease. Brain aging can lead to large differences in human lifespan, leading to age-related degenerative diseases. The potential biological age of an individual may be different from his or her actual age, and this difference can better predict the risk of age-related health problems in the future (Cole and Franke, 2017). Our model uses relaxation values alone or in combination to predict whether the brain age matches the actual age. Individuals who deviate from the brain’s aging trajectory may have unknown problems even if they are physically healthy, especially an increased risk of cognitive aging or age-related neurological diseases. Therefore, using our prediction model, we can observe the aging degree of the brain without mental and neurological abnormalities and prevent diseases in advance. Of note, the search for biomarkers of brain aging may have important neuroscience and clinical application value (Storsve et al., 2014). The brain tissue value measured by syMRI technology is characterized by objectivity, stability, good repeatability, and ease of measurement and calculation.

In this study, when exploring the relationship between brain tissue relaxation values and age, we also found that there may be differences in brain tissue relaxation values between different sexes. We found that there were differences in relaxation values between men and women in distinct brain areas, and sex differences in brain development were observed. It is not clear whether these sex differences are related to behavior. At present, studies have shown that men exhibit greater differences in brain structure than women during aging (Ritchie et al., 2018; Wierenga et al., 2018). Because there are differences in brain tissue relaxation values among different age groups, we limited the comparison between different sexes to the same age group in our study. It was found that there were differences in T1, T2, and PD values between males and females in different brain regions, and it was found that the relaxation values of most males were slightly higher than those of females. The differences between the sexes may be related to the differences in brain development between sexes. For example, in a study on the effect of sex on the development of brain executive function, it was found that girls performed significantly better than boys in reading comprehension, and boys performed better than girls in thinking during counter working memory tasks (Wierenga et al., 2019). The prevalence rates of several mental and degenerative diseases vary between sexes. For example, the incidence of Alzheimer’s disease in women is higher than that in men, prompting recent calls for biomedical research to give priority to sex differences in measures related to the disease (Mazure and Swendsen, 2016). Women also have higher rates of depression (Gennatas et al., 2017), while men have higher rates of autism spectrum disorders (Baron-Cohen et al., 2011), schizophrenia (Aleman et al., 2003) and dyslexia (Arnett et al., 2017).

SyMRI technology produces T1, T2 and PD quantitative relaxation maps simultaneously to study brain aging objectively, stably and repeatedly. Moreover, SyMRI data are easy to measure and calculate. Instead of using the cognitive score scale, which is easily affected by the subjective state of the subjects, this technique is more advantageous for exploring the biological markers of brain aging. In addition, this technique can not only be directly used for tissue quantitative analysis but can also be used for data postprocessing, such as brain tissue segmentation, brain volume quantification and myelin volume measurement (Andica et al., 2019). SyMRI can be used to quantitatively evaluate the characteristics of tissue relaxation (West et al., 2012), and this technique has been used as an objective tool to evaluate the brain development of children (Lee et al., 2018). In addition, this technique can not only be directly used for tissue quantitative analysis but can also be used for data postprocessing, such as brain tissue segmentation, brain volume quantification and myelin volume measurement (West et al., 2013; Warntjes et al., 2014).

## Conclusion

As a new MRI technique, syMRI can provide information about the changes in relaxation values in brain tissue. Our study provides specific reference ranges of T1, T2 and PD values in different brain regions from adults to elderly adults; these ranges can be used as an objective tool to evaluate brain aging. In addition, the brain age model based on syMRI is helpful in determining the degree of brain aging, which will be a useful tool to predict brain age. Different races and groups may have differences in brain structure and organization. Our study is conducted only in the Asian population, so our findings may only apply to the Asian population. Furthermore, we found that the tissue values of brain regions are different between different sexes, which provides a new way to associate sex differences in the human brain with behavior and mental illness in the future.

## Limitations

Our research has several limitations. First of all, the reference organizational values provided in this study are derived from cross-sectional data, so differences between individuals may lead to deviations in the aging process. Although the overall observations in our study are quite reliable, further longitudinal studies may be needed to confirm our findings. Secondly, the sample size of this study is relatively small, and further research is needed in a wider population to promote the clinical application of quantitative imaging using synthetic sequences. In addition, for different races and different populations, the structure and organization of the human brain will be different, our study population is only Asian people, so the results of this study may only be applicable to Asian people. Fourth, our exclusion criteria are flawed and lack of investigation of subjects’ clinical data (such as nutrition, activity, or other drugs). Finally, this study included healthy normal people who were not assessed by the Professional Mental State scale and other mental scales, and only through oral questioning of the subjects and their families without serious mental illness, learning disability and cognitive impairment in the past.

## Data availability statement

The original contributions presented in this study are included in the article/Supplementary material, further inquiries can be directed to the corresponding authors.

## Ethics statement

The studies involving human participants were reviewed and approved by the Ethics Committee of Yunnan Cancer Hospital. The patients/participants provided their written informed consent to participate in this study.

## Author contributions

SB contributed to the data analysis and article writing. CL and TK contributed to the research design. NX and AD contributed to the volunteer collection and scanning. YYL contributed to the measurement. ZO contributed to the data statistics and analysis. XG contributed to the picture and tables making. YFL contributed to the design of MRI scanning parameters. JY contributed to the research design and review of articles. All authors contributed to the article and approved the submitted version.
