# Supplementary material for: Prediction of brain age using quantitative parameters of synthetic magnetic resonance imaging
Source: Front Aging Neurosci. 2022 Nov 15;14:963668. doi: 10.3389/fnagi.2022.963668 (PMC9705592; doi:10.3389/fnagi.2022.963668)
Supplement: Supplementary file 1 [file Data_Sheet_1.docx]

Supplementary material


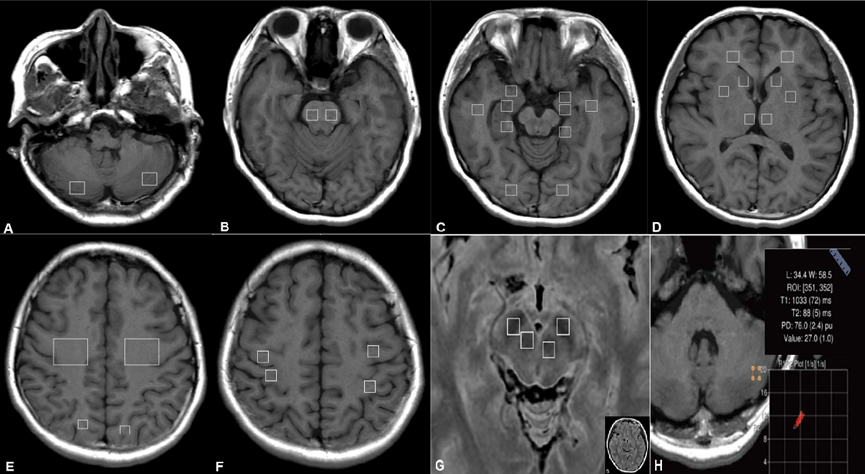


Figure 1.1：Seventeen ROIs 2D (single slice) were drawn in the cerebellar hemispheric cortex (A), pons (B), amygdala, hippocampal head, hippocampal tali, temporal lobe, occipital lobe (C), frontal lobe, caudate nucleus, putamen, dorsal thalamus (D), centrum semiovale, parietal lobe (E), precentral gyrus , postcentral gyrus (F), substantia nigra and red nucleus (G). Postprocessing software was used to extract relaxation values of brain tissue (H).


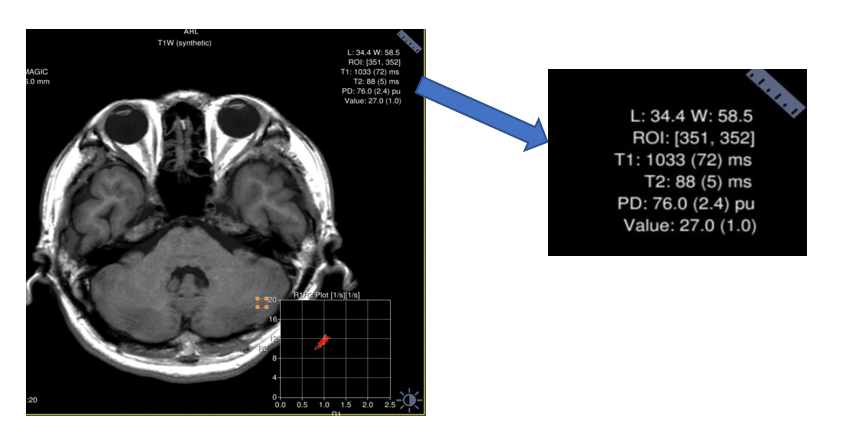


Figure 1.2：Postprocessing software was used to extract relaxation values of brain tissue

Supplementary Tables

Table 1 The ICC value of each brain region was measured twice by the observer

| ICC | T1 value | T2 value | PD value |
| --- | --- | --- | --- |
| Cerebellar cortex  Pons  Amygdala  Head of hippocanpus  Tail of hippocanpus  Temporal lobe  Occipital lobe  Frontal lobe  Caudate nucleus  Lenticular nucleus  Dorsal thalamus  Centrum semiovale  Parietal lobe  Precentral gyrus  Postcentral gyrus  Substantia nigra  Red nucleus | 0.57  0.73  0.92  0.68  0.89  0.88  0.57  0.95  0.76  0.81  0.57  0.92  0.74  0.62  0.55  0.87  0.98 | 0.70  0.76  0.89  0.78  0.95  0.82  0.74  0.95  0.92  0.93  0.84  0.94  0.80  0.72  0.73  0.93  0.94 | 0.51  0.62  0.81  0.68  0.91  0.90  0.54  0.96  0.66  0.81  0.41  0.93  0.77  0.50  0.54  0.91  0.96 |

Table 2-1 Comparison of T1 values among people of different sexes

|  | Sex | T1 value | t | P |
| --- | --- | --- | --- | --- |
| 20-30 years |  |  |  |  |
| Frontal lobe | Male（107 cases） | 1123.79±6.86 | 4.01 | ＜0.001 |
|  | Female（243 cases） | 1115.41±4.31 |  |  |
| Caudate nucleus | Male（107 cases） | 1053.89±4.38 | 4.18 | ＜0.001 |
|  | Female（243 cases） | 1033.18±2.67 |  |  |
| Lenticular nucleus | Male（107 cases） | 944.23±4.67 | 4.41 | ＜0.001 |
|  | Female（243 cases） | 921.76±2.69 |  |  |
| Centrum  semiovale | Male（107 cases） | 707.48±2.79 | 2.41 | ＜0.05 |
|  | Female（243 cases） | 699.94±1.68 |  |  |
| Substantia nigra | Male（107 cases） | 674.00±2.93 | 2.60 | ＜0.05 |
|  | Female（243 cases） | 663.94±2.53 |  |  |
| 30-40 years |  |  |  |  |
| Frontal lobe | Male（28 cases） | 639.68±5.92 | 2.72 | ＜0.01 |
|  | Female（42 cases） | 623.87±2.66 |  |  |
| 40-50 years |  |  |  |  |
| Substantia nigra | Male（33 cases） | 647.23±6.26 | 2.71 | ＜0.01 |
|  | Female（77 cases） | 629.81±3.25 |  |  |
| 50-60 years |  |  |  |  |
| Frontal lobe | Male（60 cases） | 649.76±2.97 | 2.75 | ＜0.01 |
|  | Female（110 cases） | 643.49±2.80 |  |  |
| 60-70 years |  |  |  |  |
| Frontal lobe | Male（66 cases） | 659.64±3.40 | 2.96 | ＜0.01 |
|  | Female（66 cases） | 642.59±4.64 |  |  |
| ≥70 years |  |  |  |  |
| Red nucleus | Male（63 cases） | 738.26±4.82 | 2.18 | ＜0.05 |
|  | Female（48 cases） | 723.67±4.34 |  |  |

Table 2-2 Comparison of T2 values among people of different sexes

|  | Sex | T2 values | t | P |
| --- | --- | --- | --- | --- |
| 20-30 years |  |  |  |  |
| Caudate nucleus | Male（107 cases） | 78.26±0.23 | 2.86 | ＜0.01 |
|  | Female（243 cases） | 77.48±0.15 |  |  |
| Lenticular nucleus | Male（107 cases） | 69.60±0.17 | 3.12 | ＜0.01 |
|  | Female（243 cases） | 70.59±0.27 |  |  |
| Parietal lobe | Male（107 cases） | 78.60±0.23 | -2.38 | ＜0.05 |
|  | Female（243 cases） | 79.25±0.15 |  |  |
| 30-40 years |  |  |  |  |
| Temporal lobe | Male（28 cases） | 72.54±0.62 | -2.58 | ＜0.05 |
|  | Female（42 cases） | 74.64±0.52 |  |  |
| 40-50 years |  |  |  |  |
| Hippocampal tail | Male（33 cases） | 74.36±0.52 | -2.55 | ＜0.05 |
|  | Female（77 cases） | 75.95±0.34 |  |  |
| 50-60 years |  |  |  |  |
| Pons | Male（60 cases） | 81.23±0.73 | 2.06 | ＜0.05 |
|  | Female（110 cases） | 79.66±0.39 |  |  |
| 60-70 years |  |  | — | — |
| ≥70 years |  |  | — | — |

Table 2-3 Comparison of PD values among people of different sexes

|  | Sex | PD values | t | P |
| --- | --- | --- | --- | --- |
| 20-30 years |  |  |  |  |
| Hippocampal tail | Male（107 cases） | 67.27±0.27 | -2.06 | ＜0.05 |
|  | Female（243 cases） | 67.89±0.16 |  |  |
| Frontal lobe | Male（107 cases） | 59.89±0.15 | 3.94 | ＜0.001 |
|  | Female（243 cases） | 59.16±0.10 |  |  |
| Lenticular nucleus | Male（107 cases） | 76.55±0.17 | 2.82 | ＜0.01 |
|  | Female（243 cases） | 75.97±0.11 |  |  |
| Centrum semiovale | Male（107 cases） | 62.96±0.14 | 2.47 | ＜0.05 |
|  | Female（243 cases） | 62.57±0.84 |  |  |
| 30-40 years |  |  |  |  |
| Temporal lobe | Male（28 cases） | 61.26±0.29 | 3.70 | ＜0.001 |
|  | Female（42 cases） | 59.64±0.30 |  |  |
| 40-50 years |  |  |  |  |
| Temporal lobe | Male（33 cases） | 61.02±0.27 | 3.16 | ＜0.01 |
|  | Female（77 cases） | 59.75±0.24 |  |  |
| Parietal lobe | Male（33 cases） | 62.44±0.35 | -2.28 | ＜0.05 |
|  | Female（77 cases） | 63.36±0.22 |  |  |
| 50-60 years |  |  | — | — |
| 60-70 years |  |  |  |  |
| Temporal lobe | Male（66 cases） | 62.08±0.22 | 3.16 | ＜0.01 |
|  | Female（66 cases） | 60.94±0.29 |  |  |
| Frontal lobe | Male（66 cases） | 61.45±0.25 | 3.02 | ＜0.01 |
|  | Female（66 cases） | 60.35±0.27 |  |  |
| Centrum semiovale | Male（66 cases） | 65.02±0.23 | 2.16 | ＜0.05 |
|  | Female（66 cases） | 64.37±0.20 |  |  |
| Parietal lobe | Male（66 cases） | 63.52±0.25 | -3.02 | ＜0.01 |
|  | Female（66 cases） | 64.72±0.30 |  |  |
| ≥70 years |  |  |  |  |
| Temporal lobe | Male（63 cases） | 63.29±0.20 | 2.27 | ＜0.05 |
|  | Female（48 cases） | 62.45±0.33 |  |  |

Table 3-1 Reference range of T1 values in different brain regions of different ages

| T1 value | Amygdala | Hippocampal tail | Temporal lobe | Frontal lobe | Caudate nucleus | Lenticular nucleus | Centrum semiovale | Substantia nigra | Red nucleus |
| --- | --- | --- | --- | --- | --- | --- | --- | --- | --- |
| 20-30 years (350 cases ) | 1117.97±3.65 | 726.73±2.33 | 649.69±1.57 | 628.82±1.23 | 1039.51±2.34 | 928.63±2.41 | 702.25±1.45 | 667.58±2.09 | 691.45±1.52 |
| 30-40 years（70 cases ） | 1088.19±7.12 | 708.39±4.58 | 642.09±3.38 | 630.19±2.98 | 1029.06±6.40 | 920.49±5.39 | 706.84±2.86 | 648.72±3.96 | 682.76±3.65 |
| 40-50 years（110 cases） | 1083.18±5.50 | 694.74±4.07 | 642.61±2.72 | 627.80±2.06 | 1004.55±3.99 | 909.57±4.34 | 710.84±2.26 | 635.03±3.03 | 685.62±2.80 |
| 50-60 years  （170 cases） | 1068.58±5.16 | 694.10±3.46 | 645.70±2.10 | 635.77±2.07 | 1004.41±3.20 | 923.43±3.50 | 718.62±2.08 | 631.12±2.46 | 697.48±2.44 |
| 60-70 years  （132 cases） | 1074.30±5.97 | 694.97±3.85 | 660.14±3.08 | 651.12±2.96 | 1002.10±3.73 | 940.34±5.03 | 738.84±3.08 | 639.64±2.91 | 715.61±2.78 |
| ≥70 years  （111 cases） | 1089.52±6.61 | 732.22±4.51 | 681.82±3.27 | 680.50±3.87 | 1003.07±5.21 | 972.64±5.77 | 772.01±4.38 | 652.47±3.39 | 731.95±3.37 |

Table 3-2 Reference range of T2 values in different brain regions of different ages

| T2 value | Pons | Amygdala | Hippocampal head | Hippocampal tail | Temporal lobe | Frontal lobe | Caudate nucleus |
| --- | --- | --- | --- | --- | --- | --- | --- |
| 20-30 years  (350 cases) | 82.10±0.28 | 90.97±0.30 | 94.74±0.21 | 78.71±0.17 | 74.26±0.17 | 73.01±0.14 | 77.72±0.13 |
| 30-40 years  （70 cases） | 82.24±0.68 | 87.54±0.70 | 93.07±0.47 | 76.64±0.34 | 73.80±0.42 | 72.65±0.30 | 75.46±0.30 |
| 40-50 years  （110 cases） | 81.17±0.43 | 87.63±0.44 | 93.10±0.51 | 75.48±0.29 | 74.03±0.27 | 72.98±0.25 | 74.01±0.25 |
| 50-60 years  （170 cases） | 80.21±0.37 | 87.52±0.42 | 92.32±0.33 | 75.49±0.25 | 73.93±0.26 | 72.88±0.22 | 73.03±0.24 |
| 60-70years  （132 cases） | 81.81±0.42 | 87.96±0.44 | 93.25±0.40 | 75.78±0.29 | 75.07±0.36 | 75.12±0.32 | 71.76±0.30 |
| ≥70 years  （111 cases） | 82.91±0.51 | 88.91±0.52 | 95.48±0.58 | 78.10±0.33 | 77.54±0.38 | 77.68±0.38 | 71.31±0.47 |

Table 3-3 Reference range of T2 values in different brain regions of different ages

| T2 value | Lenticular nucleus | Dorsal thalamus | Centrum semiovale | Parietal lobe | Substantia nigra | Red nucleus |
| --- | --- | --- | --- | --- | --- | --- |
| 20-30 years  (350 cases) | 69.90±0.15 | 71.29±0.16 | 82.84±0.14 | 79.05±0.13 | 61.52±0.22 | 66.30±0.19 |
| 30-40 years  （70 cases） | 66.91±0.37 | 70.74±0.33 | 82.54±0.29 | 78.08±0.29 | 59.24±0.47 | 63.84±0.39 |
| 40-50 years  （110 cases） | 64.98±0.35 | 70.07±0.33 | 83.08±0.26 | 77.56±0.24 | 57.56±0.43 | 62.65±0.38 |
| 50-60 years  （170 cases） | 63.65±0.32 | 71.03±0.27 | 83.19±0.23 | 77.22±0.20 | 57.48±0.35 | 63.16±0.35 |
| 60-70 years  （132 cases） | 63.09±0.34 | 72.72±0.34 | 85.14±0.30 | 78.31±0.26 | 57.63±0.41 | 63.42±0.38 |
| ≥70 years  （111 cases） | 63.74±0.48 | 74.36±0.34 | 88.25±0.55 | 80.06±0.30 | 59.54±0.44 | 65.68±0.43 |

Table 3-4 Reference range of PD values in different brain regions of different ages

| PD value | Amygdala | Hippocampal tail | Temporal lobe | Frontal lobe | Lenticular nucleus | Centrum  semiovale | Parietal lobe | Substantia nigra | Red nucleus |
| --- | --- | --- | --- | --- | --- | --- | --- | --- | --- |
| 20-30 years  (350 cases) | 81.83±  0.15 | 67.70±  0.14 | 60.90±  0.94 | 59.38±0.87 | 76.15±  0.96 | 62.68±  0.74 | 63.69±  0.15 | 64.31±  0.21 | 63.24±  0.11 |
| 30-40 years  （70 cases） | 81.77±  0.29 | 67.38±  0.28 | 60.29±  0.23 | 59.17±0.24 | 76.13±  0.21 | 63.00±  0.15 | 62.88±  0.23 | 63.18±  0.47 | 62.70±  0.23 |
| 40-50 years  （110 cases） | 81.41±  0.22 | 67.03±  0.22 | 60.13±  0.19 | 59.17±0.15 | 75.54±  0.20 | 63.17±  0.12 | 63.08±  0.19 | 63.82±  0.37 | 63.22±  0.23 |
| 50-60 years  （170 cases） | 81.08±  0.21 | 66.85±  0.18 | 60.59±  0.15 | 60.00±0.13 | 76.18±  0.17 | 63.57±  0.99 | 63.23±  0.18 | 63.25±  0.29 | 63.78±  0.16 |
| 60-70 years  （132 cases） | 81.22±  0.24 | 67.15±  0.23 | 61.51±  0.19 | 60.90±0.19 | 78.98±  1.90 | 64.69±  0.15 | 64.12±  0.20 | 64.27±  0.27 | 64.88±  0.20 |
| ≥70 years  （111 cases） | 81.40±  0.28 | 69.29±  0.24 | 62.92±  0.19 | 62.54±0.21 | 77.78±  0.23 | 66.06±  0.18 | 65.55±  0.27 | 64.23±  0.36 | 66.34±  0.23 |

Table 4-1 Spearman correlation coefficient between T1 value of each brain region and age

| Brain region | P | r |
| --- | --- | --- |
| Amygdala | ＜0.001 | -0.240^**^ |
| Hippocampal tail | ＜0.001 | -0.163^**^ |
| Temporal lobe | ＜0.001 | 0.218^**^ |
| Frontal lobe | ＜0.001 | 0.376^**^ |
| Caudate nucleus | ＜0.001 | -0.347^**^ |
| Lenticular nucleus | ＜0.001 | 0.148^**^ |
| Centrum semiovale | ＜0.001 | 0.513^**^ |
| Substantia nigra | ＜0.001 | -0.259^**^ |
| Red nucleus | ＜0.001 | 0.343^**^ |

Table 4-2 Spearman correlation coefficient between T2 value of each brain region and age

| Brain region | P | r |
| --- | --- | --- |
| Pons | ＞0.05 | — |
| Amygdala | ＜0.001 | -0.216^**^ |
| Hippocampal head | ＜0.05 | -0.103^**^ |
| Hippocampal tail | ＜0.001 | -0.276^**^ |
| Temporal lobe | ＜0.001 | 0.164^**^ |
| Frontal lobe | ＜0.001 | 0.298^**^ |
| Caudate nucleus | ＜0.001 | -0.659^**^ |
| Lenticular nucleus | ＜0.001 | -0.628^**^ |
| Dorsal thalamus | ＜0.001 | 0.179^**^ |
| Centrum semiovale | ＜0.001 | 0.343^**^ |
| Parietal lobe | ＞0.05 | — |
| Substantia nigra | ＜0.001 | -0.289^**^ |
| Red nucleus | ＜0.001 | -0.216 |

Table 4-3 Spearman correlation coefficient between PD value of each brain region and age

| Brain region | P | r |
| --- | --- | --- |
| Amygdala | ＜0.05 | -0.109^**^ |
| Hippocampal tail | ＞0.05 | — |
| Temporal lobe | ＜0.001 | 0.194^**^ |
| Frontal lobe | ＜0.001 | 0.377^**^ |
| Lenticular nucleus | ＜0.001 | 0.195^**^ |
| Centrum semiovale | ＜0.001 | 0.544^**^ |
| Parietal lobe | ＜0.001 | 0.118^**^ |
| Substantia nigra | ＞0.05 | — |
| Red nucleus | ＜0.001 | 0.366^**^ |

Table 5-1 Variables screened by Logistic binary regression analysis（T1 values）

|  | OR（95%CI） | P |
| --- | --- | --- |
| Caudate nucleus | 0.986（0.980-0.992） | ＜0.001 |
| Lenticular nucleus | 1.009（1.003-1.015） | ＜0.05 |
| Centrum semiovale | 1.019（1.011-1.028） | ＜0.001 |
| Substantia nigra | 0.998（0.982-0.995） | ＜0.05 |
| Red nucleus | 1.023（1.015-1.028） | ＜0.001 |

Table 5-2 Variables screened by Logistic binary regression analysis（T2 values）

|  | OR（95%CI） | P |
| --- | --- | --- |
| Frontal lobe | 1.204（1.114-1.302） | ＜0.001 |
| Caudate nucleus | 0.781（0.708-0.861） | ＜0.001 |
| Lenticular nucleus | 0.907（0.842-0.997） | ＜0.05 |
| Dorsal thalamus | 1.285（1.193-1.384） | ＜0.001 |

Table 5-3 Variables screened by Logistic binary regression analysis（PD values）

|  | OR（95%CI） | P |
| --- | --- | --- |
| Centrum semiovale | 1.912（1.629-2.244） | ＜0.001 |
| Red nucleus | 1.22（1.118-1.331） | ＜0.001 |
